# Infective Endocarditis Causing Acute Myocardial Infarction

**DOI:** 10.7759/cureus.11245

**Published:** 2020-10-29

**Authors:** Seth Cohen, Lucie Ford, Elaine Situ-LaCasse, Noah Tolby

**Affiliations:** 1 Emergency Medicine, University of Arizona, Tucson, USA; 2 Emergency Medicine, Eastern Virginia Medical School, Norfolk, USA

**Keywords:** endocarditis, septic pulmonary embolism, stemi, pocus

## Abstract

Endocarditis is a well-known disease, yet septic embolization resulting in myocardial infarction is much rarer and very infrequently diagnosed in the emergency department (ED). Point-of-Care-Ultrasound (POCUS) can be used to confirm clinical suspicion within minutes of patient presentation, thereby expediting patient care.

We report the case of a 26-year-old female with known intravenous drug use who presented with altered mental status. Her clinical presentation prompted urgent evaluation in the ED with POCUS which showed a hyperdynamic functioning left ventricle, greater than 50% inferior vena cava collapse, and a large tricuspid valve vegetation. In light of the electrocardiogram (ECG) ST changes suggesting an acute myocardial infarction, the patient was emergently taken to the cardiac catheterization laboratory where coronary angiography revealed multiple coronary emboli. Primary diagnoses included endocarditis due to* Staphylococcus*, septic pulmonary embolism, and ST-elevated myocardial infarction (STEMI) due to embolic occlusion of the distal left anterior descending artery.

Myocardial infarction caused by septic embolization from endocarditis is a rare condition; however, POCUS is a quick, non-invasive tool that can aid the emergency medicine (EM) physician in identifying this life-threatening pathology thereby expediting appropriate care for the patient.

## Introduction

Infectious endocarditis (IE) is well known to cause complications such as congestive heart failure, periannular abscesses, and systemic embolization. While this embolization most commonly affects the central nervous system [1], septic embolization resulting in acute coronary syndrome has an incidence of 2.2% [2], with a significant mortality rate of approximately 64% [3]. Type one myocardial infarctions (MI), caused by acute atherothrombotic events, cause approximately 97% of myocardial infarctions [4] and have a well-defined intervention and management pathway. Type two myocardial infarctions are comprised of ischemia not due to coronary artery disease, but rather due to a mismatch in myocardial oxygen supply and demand [5]. While emboli from endocarditis vegetations causing an acute MI are rare, recognition and prompt treatment are crucial due to the high mortality rate and additionally help to direct interventions as MI caused by septic emboli are treated differently than a typical MI [6]. Point-of-Care-Ultrasound (POCUS) use in the ED can help distinguish the MI cause, ultimately resulting in faster treatment and lower mortality. 

## Case presentation

A 26-year-old female was transported to the ED by Emergency Medical Services (EMS) for an altered level of consciousness of unknown onset. Her past medical history as obtained from EMS personnel on scene was significant for intravenous drug use (IVDU) and possible recent elective abortion. On presentation to the ED, patient was alert to name only and very agitated. She had a temperature of 40°C (104°F), a heart rate of 160 regular beats/min, a blood pressure of 88/55 mm Hg, a respiratory rate of 41 breaths/min, and a pulse oximetry reading of 88% on room air. Patient was pale, auscultation of the lungs were coarse bilaterally, heart sounds did not reveal any murmurs, rubs or gallops, and heavy vaginal bleeding was noted. 

**Figure 1:**
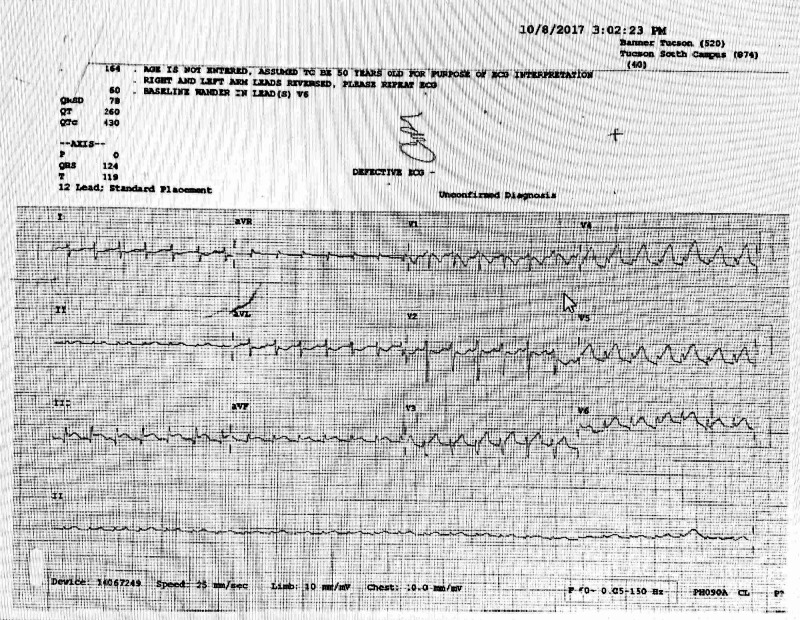
Patient's initial electrocardiogram depicting ST elevations

Initial stabilization and evaluation included supplemental oxygen, intravenous fluid resuscitation, an electrocardiogram (ECG) and POCUS echocardiogram. Blood and urine were sent to the laboratory as well. Her ECG (Figure 1) showed sinus tachycardia at 164 beats/min, a rightward axis deviation (RAD), 1-mm ST elevation in Lead II, 3-mm ST elevation in Lead III, 2-mm ST elevation in Lead aVF, V4-V6, with reciprocal changes in I and aVL consistent with an acute inferior-lateral injury pattern. The presence of ST elevations along with RAD, hypoxia and the patient’s shocky state supported pulmonary embolus remaining high on the differential. Other differential diagnoses considered included septic shock, cardiogenic shock, and complications of a recent elective abortion.

**Figure 2:**
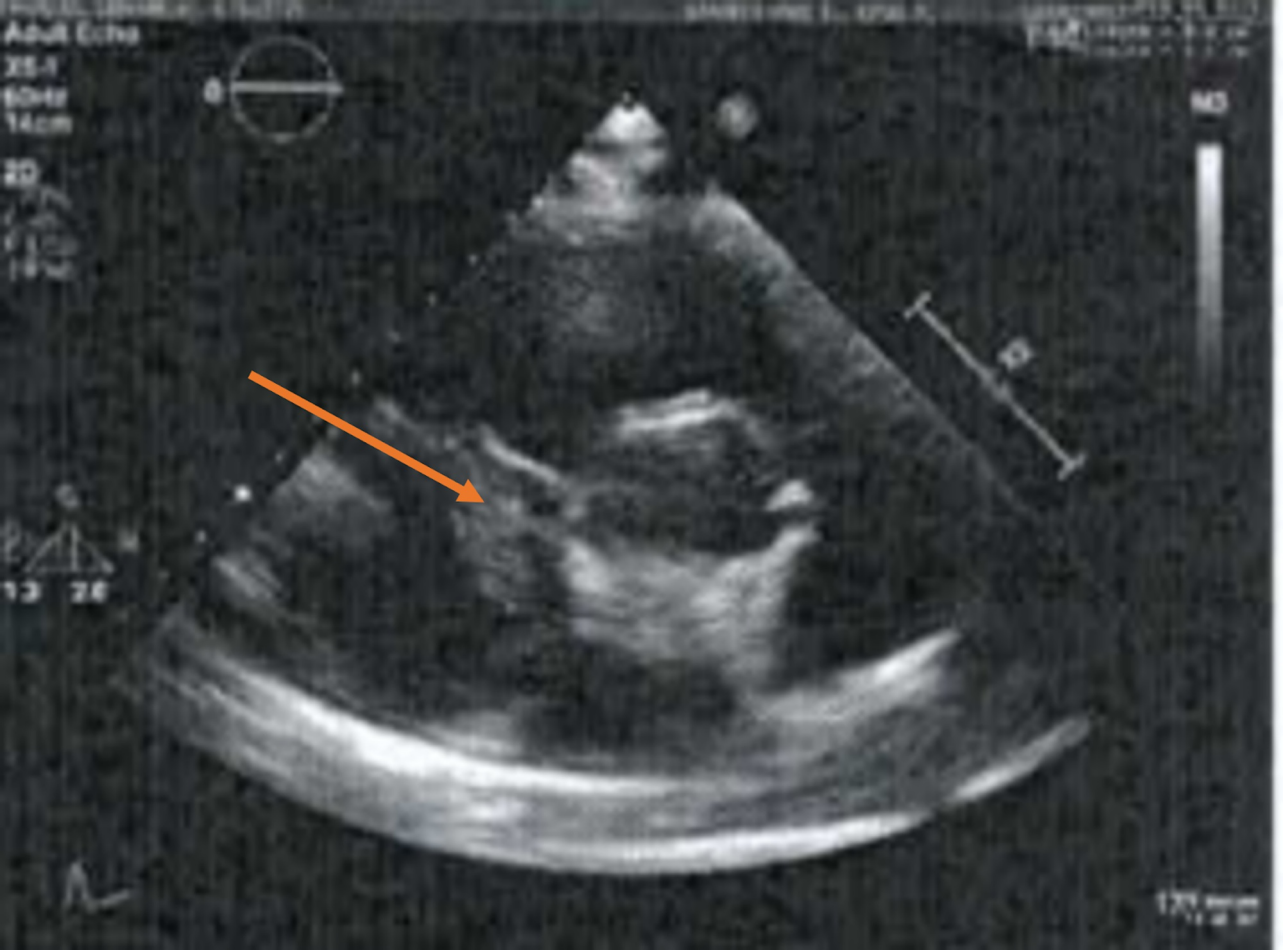
POCUS in the emergency department showing tricuspid valve vegetation POCUS- Point of care ultrasound

An ST-elevated myocardial infarction (STEMI) alert was called. POCUS echocardiogram showed a hyperdynamic functioning left ventricle, a greater than 50% inferior vena cava (IVC) collapse, no cardiac tamponade, no pericardial effusion, and no RV dilatation, thus a large pulmonary embolism was considered less likely. A large tricuspid valve vegetation was noted (Figure 2), and in this patient without known cardiovascular disease or risk factors, embolic occlusion of a coronary artery from the valvular mass was the most likely explanation of the ECG findings. 

Initial pertinent laboratory results returned with white blood cells (WBCs) of 18,300 with 91% polymorphonuclear leukocytes (PMN), hemoglobin 8.6, hematocrit 25, platelets 14,000, prothrombin time (PT) 16.5, international normalized ratio (INR) 1.5, human chorionic gonadotropin (hCG) quant 474, lactic acid of 3.1 and initial troponin of 23.28. Electrolytes were all within normal limits, carbon monoxide (CO) of 17, blood urea nitrogen (BUN) of 43 and creatinine of 1.16. Liver function tests revealed abnormalities of total protein of 5, albumin 1.9, total bilirubin 3.2, alkaline phosphatase 474, and creatine phosphokinase of 356. Urine drug screen was positive for amphetamines and opiates. Ammonia was normal at 30. Portable chest x-ray was interpreted as multifocal pneumonia versus showered septic emboli. 

ED administration of antiplatelet and antithrombotic therapy was withheld in consultation with cardiology due to the patient’s thrombocytopenia and active vaginal bleeding and she was taken emergently to the cardiac cath laboratory. Coronary angiography revealed a 100% distal left anterior descending occlusion with no distal flow and a 100% occlusion of a small distal segment of the right posterior descending artery, noted by cardiology to be a very small vessel. Thrombus aspiration of the left anterior descending artery followed by balloon angioplasty restored flow. Patient did receive a pre-catheterization heparin bolus and intracoronary abciximab and nicardipine after flow was reestablished. The remaining coronary arteries were angiographically normal and patent. Dual anti-platelet therapy was held until a head CT could be obtained to rule out intracranial hemorrhage. Head CT showed several areas of hyperdensity in the left frontoparietal junction and right frontal lobe consistent with likely subarachnoid hemorrhages stemming from septic emboli. Formal echocardiogram showed apical akinesis, mitral valve vegetation of the posterior leaflet measuring 0.6 cm x 0.5 cm with mild mitral regurgitation, and a tricuspid valve vegetation on the anterior leaflet measuring 2.4 cm x 1.3 cm with severe tricuspid regurgitation. The right ventricle pressure was elevated to 60 mmHg plus central venous pressure and tricuspid annular plane systolic excursion measured 18mm. There was mild right atrial dilation, global right ventricle size was mildly increased and mild pulmonary regurgitation was noted. 

**Figure 3:**
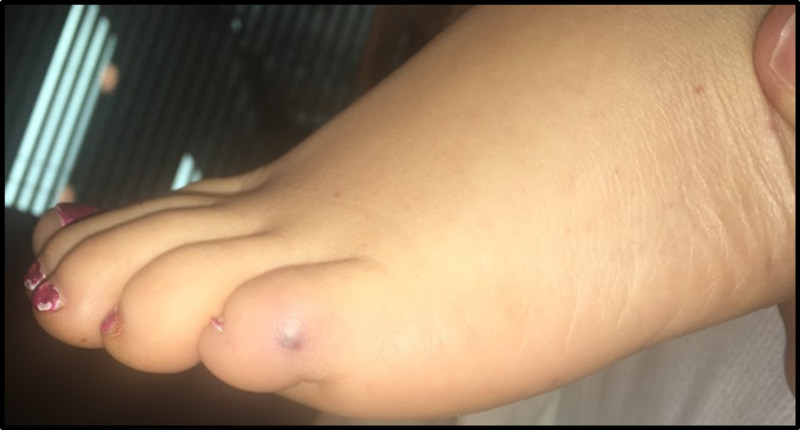
Janeway lesion

**Figure 4:**
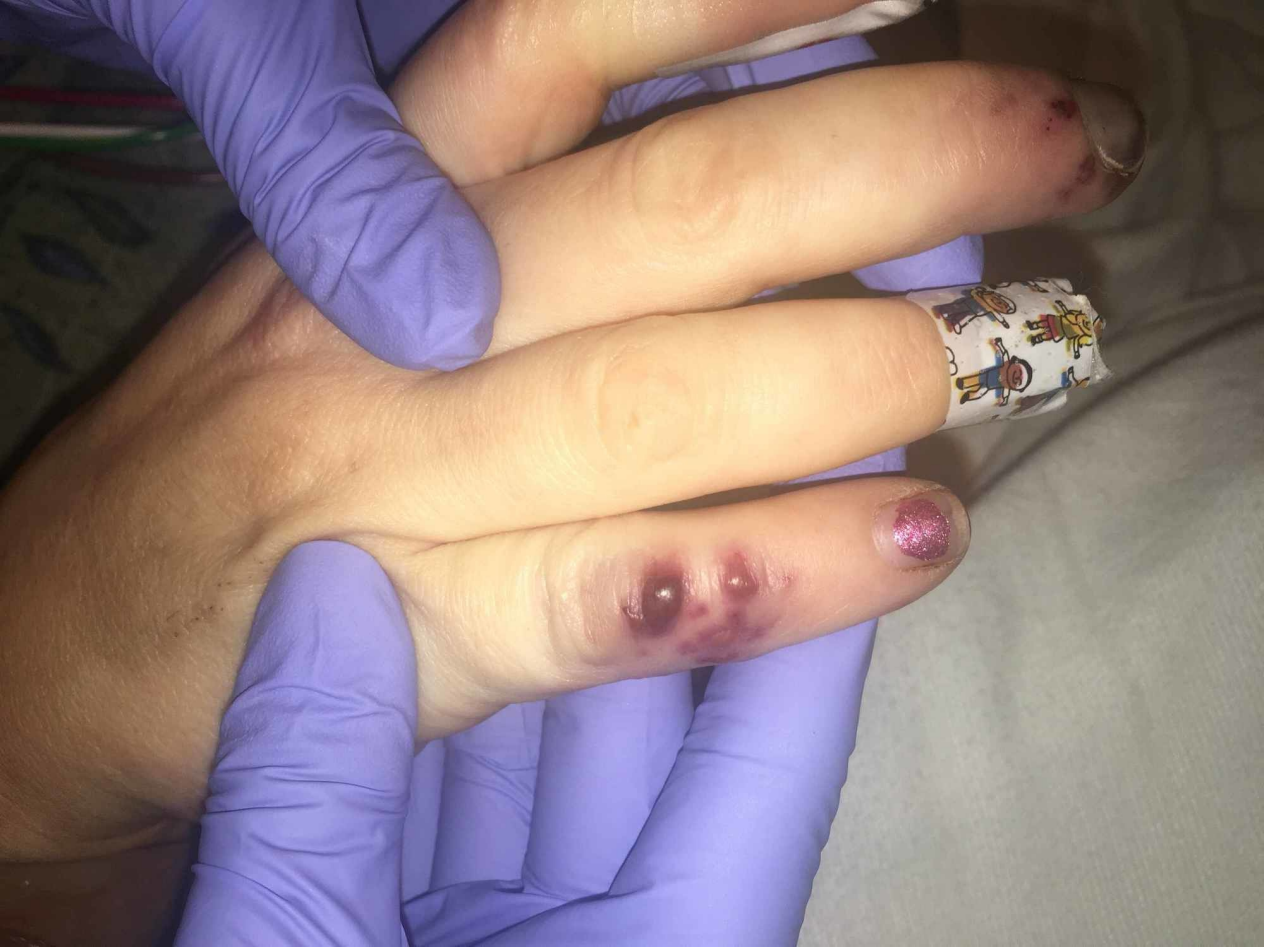
Osler's nodes

On examination of the patient in the intensive care unit, the classic physical skin findings of bacterial endocarditis were more apparent with the presence of splinter hemorrhages under her nails, Janeway lesions on the soles of her feet and toes bilaterally (Figure 3), and Osler’s nodes on her hands. The most severe were noted to her right fourth and fifth digits which eventually became necrotic (Figure 4).

After cardiac catheterization, the patient remained thermodynamically labile with altered mental status, therefore was electively intubated and transferred to a higher-level care facility where cardiothoracic surgical services were available. Initial blood cultures grew methicillin-sensitive *Staphylococcus aureus* and the patient remained on pressors and antibiotics for several weeks. Ultimately the patient required continuous renal replacement therapy and a tracheostomy. 

## Discussion

Hospitalizations for IE from IVDU have increased from 7% in 2000 to 12.1% in 2013, although these numbers may be underreported as the system referenced captured ICD-9 codes [7]. A single-center chart review study showed hospitalizations for IE from IVDU increased from 14% in 2009 to 56% in 2016 [8]. In addition to IE from IVDU, the incidence of IE from implanted cardiac devices has also increased as more of these devices are being deployed [9]. Coronary embolization is a very uncommon complication of IE at approximately 7%. While prevalence is low, this disease carries a high short- and long-term mortality and morbidity rate and therapeutic strategies for management are controversial [4,6,10].

Septic emboli from indwelling catheters or patients on dialysis have been reported [11], yet few reports of STEMI from acute infective endocarditis secondary to IVDU are found in the literature. 

Management of these patients presents unique challenges. Percutaneous coronary intervention is feasible to facilitate aspiration thrombectomy, yet this may not be sufficient and may even lead to more embolizations, particularly when vegetations are present on the aortic valve. Angioplasty or stent placement during percutaneous coronary intervention (PCI) is also risky as it can lead to distal septic embolization or aneurysm formation at the site [6]. Thrombolytics are not an ideal treatment as the emboli are not atherosclerotic in origin so lysis is not likely to have an effect. Additionally, a significant percentage of patients (65%) will have cerebral emboli and IV thrombolytics greatly increase the risk of cerebral hemorrhage [12].

## Conclusions

POCUS use in the ED is a quick, non-invasive tool that can aid the EM physician in identifying life-threatening pathology and expediting care for the patient. 
